# Successful Omalizumab treatment in delayed pressure urticaria as confirmed by stimulation test^☆^

**DOI:** 10.1016/j.abd.2024.06.008

**Published:** 2025-01-10

**Authors:** Cesare Ariasi, Gabriele Perazzolli, Luca Rubelli, Alessandro Fraghì, Piergiacomo Calzavara-Pinton

**Affiliations:** Department of Dermatology, ASST Spedali Civili di Brescia, University of Brescia, Brescia, Italy

*Dear Editor,*

Delayed Pressure Urticaria (DPU) is a rare form of Chronic Inducible Urticaria (CIndUs) defined by the appearance of a skin swelling response after the application of a sustained pressure stimulus to the skin.^1^

DPU is chronic and it can be profoundly disabling, causing painful and prolonged swelling of feet and hands, as well as systemic symptoms of malaise and flu-like illness. It is often a treatment challenge because of its resistance to H_1_-antihistamines and Leukotriene antagonist.^2^ Nowadays, Omalizumab allows us to obtain a complete remission in patients with mixed Chronic spontaneous urticaria associated with DPU.^3^ Moreover, omalizumab has been used off-label for the treatment of DPU not associated with CSU. Omalizumab efficacy has already been reported in DPU,^4^ but confirmatory stimulation retesting with threshold determination has not been reported yet.

We report the case of a 35-year-old man with a 3-year history of severe DPU. Important swelling at the palms and soles that deeply impaired working and leisure activities. The patient had no evidence of any underlying disease. Therefore, he was treated with cetirizine at a four-fold daily licensed dose, Montelukast 10 mg/day, systemic corticosteroids, Cyclosporin 4 mg/Kg/day, showing only mild improvement of the disease. Complete remission of symptoms was only obtained with 0.5 mg/kg/day of prednisone. However, re-exacerbation of symptoms occurred during the tapering of the prednisone daily dose. Therefore, we decided to treat the patient with Omalizumab at the dosage of 300 mg every 4 weeks.

Before starting the anti-IgE therapy the patient’s total IgE levels were 159 U/mL. Blood count, TSH, Anti-TPO antibodies and Anti-TG antibodies were in range of normality. *H. pylori* stool antigen assay was negative. The Urticaria Activity Score for the preceding 7 days (UAS7) and Urticaria Control Test (UCT) scores were respectively 26 (moderate to severe disease activity) and 6 (poorly controlled urticaria).^5^ Pressure test was positive (Fig. 1A‒B), urticarial reaction appeared 4 hrs after the end of provocation testing associated with malaise, “flu-like” symptoms, and arthralgias (Fig. 1B). The test was performed by adapting the criteria from the Charité Hospital,^6^ administering a pressure of 20.7 kPa for 15 minutes using a sphygmomanometer maintained at a constant pressure of around 155 mmHg (Fig. 1A), equivalent to 20.7 kPa (In accordance with the equivalence of 1 kPa to 7.50062 mmHg). After the first dose of Omalizumab, the patient experienced a progressive resolution of the symptoms with complete remission after 14 days. Retesting was performed after three months of therapy with the same protocol (Fig. 2A). Provocation test was negative during the 24 hrs observation time (Fig. 2B). Therefore, we looked for a threshold of provocation, but repeated stimulations at higher pressures of 24 kPa (equivalent to 180 mmHg) did not elicit any response. UAS7 and UCT scores,^5^ performed every month from the start of the therapy, were constantly 0 (no disease activity) and 15 (well-controlled urticaria). For the excellent clinical response achieved Omalizumab was interrupted after 6 months of therapy. However, the relapse after 2 months led to start a second cycle of Omalizumab at the same dosage with a complete response after 2 weeks.

**Figure 1:**
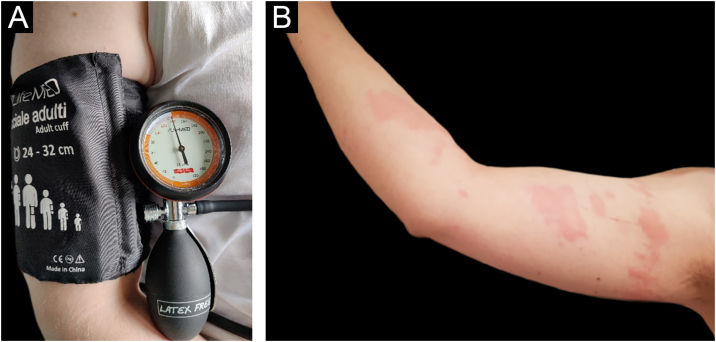
(A) Provocation test performed using a forearm sling with a pressure of 20.7 kPa for 15 min. (B) Patient’s right arm, 4 hours after provocation test using a forearm sling with a pressure of 20.7 kPa for 15 min during therapy with Cetirizine at a four-fold daily dose. The patient reported multiple diffuse skin swelling associated with nausea and chills.

**Figure 2:**
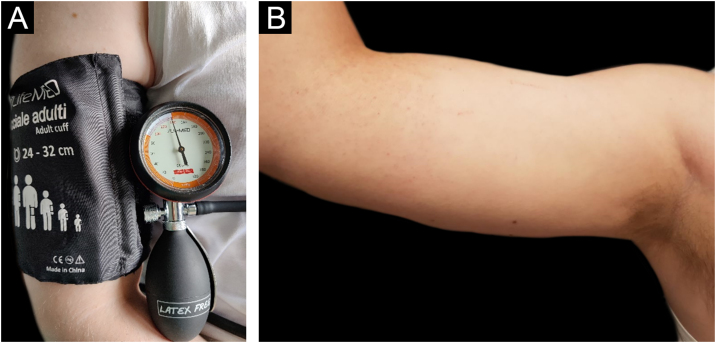
(A) Provocation test performed using a forearm sling with a pressure of 20.7 kPa for 15 min. (B) Patient’s right arm, 4 hours after provocation test using a forearm sling with a pressure of 20.7 kPa for 15 min after 3 administrations of Omalizumab 300 mg every 4 weeks. Negative for skin lesions and systemic symptoms.

In conclusion in our case Omalizumab remarked its efficacy in DPU, preventing cutaneous manifestation and systemic symptoms, even though this drug is not licensed for CIndUs. The pathogenesis of DPU is not well characterized, although a number of potential mechanisms and mediators have been postulated. The efficacy of IgE inhibition in this report supports the role of mast cell activation in pressure-induced wheals. Further studies are necessary to support the evidence that omalizumab is effective in preventing symptoms in DPU. However, in order to better compare the results, we encourage the use of standardized and harmonized tests protocols, as suggested by EAACI guidelines.^6^

## Financial support

None declared.

## Authors’ contributions

Cesare Ariasi: Contributed to data collection; analysis and interpretation; writing of the manuscript; critical review of the literature and final approval of the final version of the manuscript and data collection.

Gabriele Perazzolli: Contributed to intellectual participation in the propaedeutic and/or therapeutic conduct of the studied cases; final approval of the final version of the manuscript and data collection.

Luca Rubelli: Contributed to data collection; writing of the manuscript and final approval of the final version of the manuscript and data collection.

Alessandro Fraghì: Contributed to data collection; writing of the manuscript and final approval of the final version of the manuscript and data collection.

Piergiacomo Calzavara-Pinton: Contributed to the study concept and design, to the intellectual participation in the propaedeutic and/or therapeutic conduct of the studied cases and final approval of the final version of the manuscript and data collection.

## Conflicts of interest

None declared.
